# Assessment of the safety of Roxadustat for cardiovascular events in chronic kidney disease-related anemia using meta-analysis and bioinformatics

**DOI:** 10.3389/fphar.2024.1380326

**Published:** 2024-06-19

**Authors:** Xiangmeng Li, Shimin Jiang, Xia Gu, Xiaojing Liu, Shunlai Shang, Jiao Zhang, Keying Pang, Wenge Li

**Affiliations:** ^1^ Department of Nephrology, China-Japan Friendship Hospital, Beijing, China; ^2^ Peking Union Medical College, Beijing, China; ^3^ Key Laboratory of Chronic Kidney Disease and Mineral Metabolism of Hebei Province, Baoding, Hebei, China; ^4^ Beijing University of Chinese Medicine, Beijing, China; ^5^ College of Pharmacy, Hebei University of Chinese Medicine, Shijiazhuang, Hebei, China

**Keywords:** Roxadustat, erythropoietin stimulating agents, cardiovascular events, meta-analysis, inflammatory responses

## Abstract

**Objective:**

This study compares the cardiovascular risk in anemic chronic kidney disease patients treated with Roxadustat versus erythropoietin stimulating agents (ESAs). It also explores the cardiovascular impact of Roxadustat.

**Methods:**

We searched PubMed, EMBASE, Cochrane, Scopus, and Web of Science databases up to 13 August 2023, using terms such as “ESA,” “Roxadustat,” “MACE,” “stroke,” “death,” “myocardial infarction,” and “heart failure.” Two researchers independently selected and extracted data based on predefined criteria. We assessed the risk of bias with the Cochrane tool and analyzed statistical heterogeneity using the Q and I2 tests. We conducted subgroup analyses by geographical region and performed data analysis with Stata 14.0 and RevMan 5.4 software. Data were sourced from the NCBI database by filtering for “Roxadustat” and “human,” and differentially expressed genes were identified using R software, setting the significance at *p* < 0.01 and a 2-fold logFC, followed by GO enrichment analysis, KEGG pathway analysis, and protein interaction network analysis.

**Results:**

A total of 15 articles encompassing 1,43,065 patients were analyzed, including 1,38,739 patients treated with ESA and 4,326 patients treated with Roxadustat. In the overall population meta-analysis, the incidences of Major Adverse Cardiovascular Events (MACE), death, and heart failure (HF) were 13%, 8%, and 4% in the Roxadustat group, compared to 17%, 12%, and 6% in the ESA group, respectively, with *P*-values greater than 0.05. In the subgroup analysis, the incidences were 13%, 11%, and 4% for the Roxadustat group versus 17%, 15%, and 5% for the ESA group, also with *p*-values greater than 0.05. Bioinformatics analysis identified 59 differentially expressed genes, mainly involved in the inflammatory response. GO enrichment analysis revealed that these genes are primarily related to integrin binding. The main pathways identified were the TNF signaling pathway, NF-κB signaling pathway, and lipid metabolism related to atherosclerosis. The protein interaction network highlighted IL1B, CXCL8, ICAM1, CCL2, and CCL5 as the top five significantly different genes, all involved in the inflammatory response and downregulated by Roxadustat, suggesting a potential role in reducing inflammation.

**Conclusion:**

The meta-analysis suggests that the use of Roxadustat and ESA in treating anemia associated with chronic kidney disease does not significantly alter the likelihood of cardiovascular events in the overall and American populations. However, Roxadustat exhibited a safer profile with respect to MACE, death, and heart failure. The bioinformatics findings suggest that Roxadustat may influence integrin adhesion and affect the TNF and NF-κB signaling pathways, along with lipid and atherosclerosis pathways, potentially reducing inflammation.

## Graphical Abstract

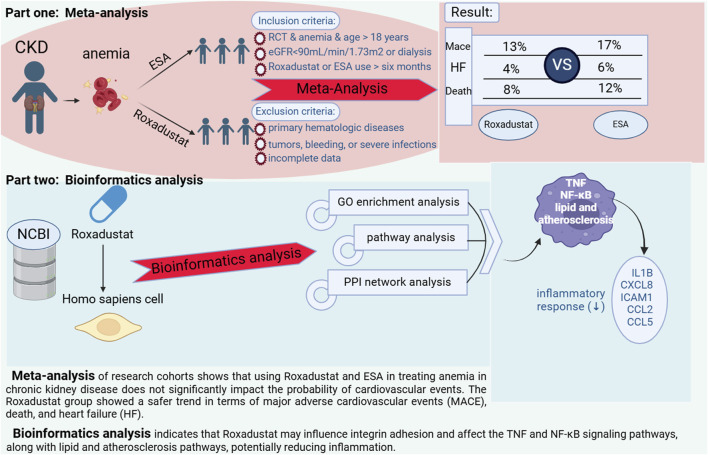

## 1 Introduction

Anemia in patients with chronic kidney disease (CKD) often results from reduced erythropoietin (EPO) production, leading to lower levels of red blood cells and hemoglobin. This condition significantly impairs patient health and functionality, evident through symptoms such as fatigue, weakness, palpitations, and dizziness, which in turn increase mortality risk. Managing anemia in CKD patients is essential for enhancing their quality of life and prognosis. Erythropoiesis-stimulating agents (ESAs) are crucial for providing supplemental EPO through injections. However, many patients show poor compliance with ESA treatment, which is a major factor in anemia management failure. It is reported that non-compliance, defined as taking less than 90% of the prescribed dose, affects 35%–55% of the dialysis population (Johnson et al., 2007). The need for cold chain transportation for ESAs also adds significant economic burdens. Roxadustat, the first hypoxia-inducible factor prolyl hydroxylase inhibitor (HIF-PHI), has shown promise in improving anemia (Provenzano et al., 2021a; Provenzano et al., 2021b; Charytan et al., 2021; Fishbane et al., 2022) and is administered orally, which could enhance patient compliance. Nevertheless, its cardiovascular safety remains controversial. This study aims to assess the risk of cardiovascular events in CKD patients with anemia treated with Roxadustat through a two-part approach: a meta-analysis and bioinformatics analysis. The first part will conduct a meta-analysis of cardiovascular events in these patients to evaluate Roxadustat’s safety, registered under CRD42023453280 on the PROSPERO website. The second part will utilize GO enrichment analysis, pathway analysis, and protein interaction network analysis of RNAseq data from human cells treated with Roxadustat, available in the NCBI database, to investigate the drug’s regulatory mechanisms on cardiovascular events.

## 2 Materials and methods

### 2.1 Meta-analysis of cardiovascular events in chronic kidney disease anemia patients treated with Roxadustat

#### 2.1.1 Search strategy

The PubMed, EMBASE, Cochrane, Scopus, and Web of Science databases were searched or studies using the search terms “ESA, Roxadustat, MACE, stroke, death, myocardial infarction, heart failure” up to 13 August 2023.

#### 2.1.2 Inclusion and exclusion criteria

Inclusion criteria included (Johnson et al., 2007) randomized controlled trials; (Provenzano et al., 2021a); patients with chronic kidney disease (eGFR<90 mL/min/1.73 m^2^) including those on dialysis; (Provenzano et al., 2021b); anemia (Hb<10 g/L); (Charytan et al., 2021); participants aged over 18 years; (Fishbane et al., 2022); use of roxadustat or ESA for at least 6 months.

Exclusion criteria included (Johnson et al., 2007) anemia due to primary hematologic diseases; (Provenzano et al., 2021a); anemia secondary to tumors, bleeding, or severe infections; (Provenzano et al., 2021b); study populations with conditions other than chronic kidney disease, such as those with concurrent diabetes; (Charytan et al., 2021); incomplete data, even after contacting study authors, making certain indicators unavailable. Two authors independently screened the literature and extracted data to minimize selection bias.

#### 2.1.3 Literature screening and data extraction

Data were independently extracted by two authors from the selected literature. In case of discrepancies, a third author participated in the extraction process, and data were reported to the corresponding author of this study. Extracted data included the original geographical region of the study population (Asia, America, Europe), publication year, sample size, total follow-up time, eGFR levels and dialysis status, gender, average age, medical history, average hemoglobin levels, types and doses of medications, and cardiovascular events. Cardiovascular events comprised MACE and heart failure, with MACE including death, myocardial infarction, and stroke. The medical history primarily involved cardiac and cerebrovascular diseases. Cardiovascular events were the outcome events of this study. Differences in reporting led to treating death and all-cause mortality as equivalent, as well as acute myocardial infarction, myocardial infarction, and non-fatal myocardial infarction as equivalent, and similarly for stroke events and non-fatal stroke events.

#### 2.1.4 Methodological quality assessment

This study applied the Cochrane risk of bias assessment tool, evaluating whether (Johnson et al., 2007) randomization methods introduced bias; (Provenzano et al., 2021a); allocation methods introduced bias; (Provenzano et al., 2021b); blinding was implemented; (Charytan et al., 2021); data results were complete; (Fishbane et al., 2022); study results introduced bias; (Chertow et al., 2021); other sources of bias were present. If all criteria were met, the risk of bias was considered low; if some were met, the risk was moderate; if none were met, the risk was high. The study excluded literature with a high risk of bias and conducted bias testing and sensitivity analysis on the results.

#### 2.1.5 Data analysis

Johnson et al. (2007) Compare the risk of cardiovascular events between chronic kidney disease anemia patients treated with Roxadustat and those treated with ESA across the overall population (Provenzano et al., 2021a); Group the study population by geographic region in cohort studies to compare the risk of cardiovascular events post-treatment with Roxadustat and ESA. As cardiovascular events associated with Roxadustat were reported exclusively in the Americas, the analysis focused only on American patients (Provenzano et al., 2021b); Heterogeneity Test: Statistical heterogeneity of the included studies was analyzed using the Q test and the I^2^ test. An I^2^ value less than 50% indicates moderate heterogeneity, while values above 50% indicate high heterogeneity. A fixed-effect model is used when there is no statistical heterogeneity; otherwise, a random-effects model is employed (Charytan et al., 2021); Funnel plots were utilized to evaluate publication bias (Fishbane et al., 2022); Stata 14.0 software and RevMan 5.4 were employed for the meta-analysis.

### 2.2 Bioinformatic analysis of the effects of Roxadustat on human cells

#### 2.2.1 Data source

The dataset was sourced using “Roxadustat” and “human (*Homo sapiens*)” as search terms in the NCBI database.

#### 2.2.2 Data processing

The selected dataset underwent preprocessing and analysis with R software, focusing on identifying differentially expressed genes based on a threshold of *p* < 0.01 and a 2-fold log fold change (logFC).

#### 2.2.3 GO enrichment analysis and KEGG pathway analysis

Initially, GO enrichment analysis was conducted to understand the biological processes, cellular components, and molecular functions of the differentially expressed genes using data from the GO database. This was followed by KEGG pathway enrichment analysis to explore the cellular signaling and biological metabolic pathways of the genes. All results from these analyses were required to meet a significance level of *p* < 0.05.

#### 2.2.4 Building protein-protein interaction (PPI) network

The protein-protein interaction network for differentially expressed genes was constructed using the STRING database and Cytoscape software to delineate interactions and identify potential key regulators influenced by roxadustat.

## 3 Results

### 3.1 Meta-analysis of Roxadustat for the treatment of anemia in chronic kidney disease patients with cardiovascular events

#### 3.1.1 Research search and screening

**FIGURE 1:**
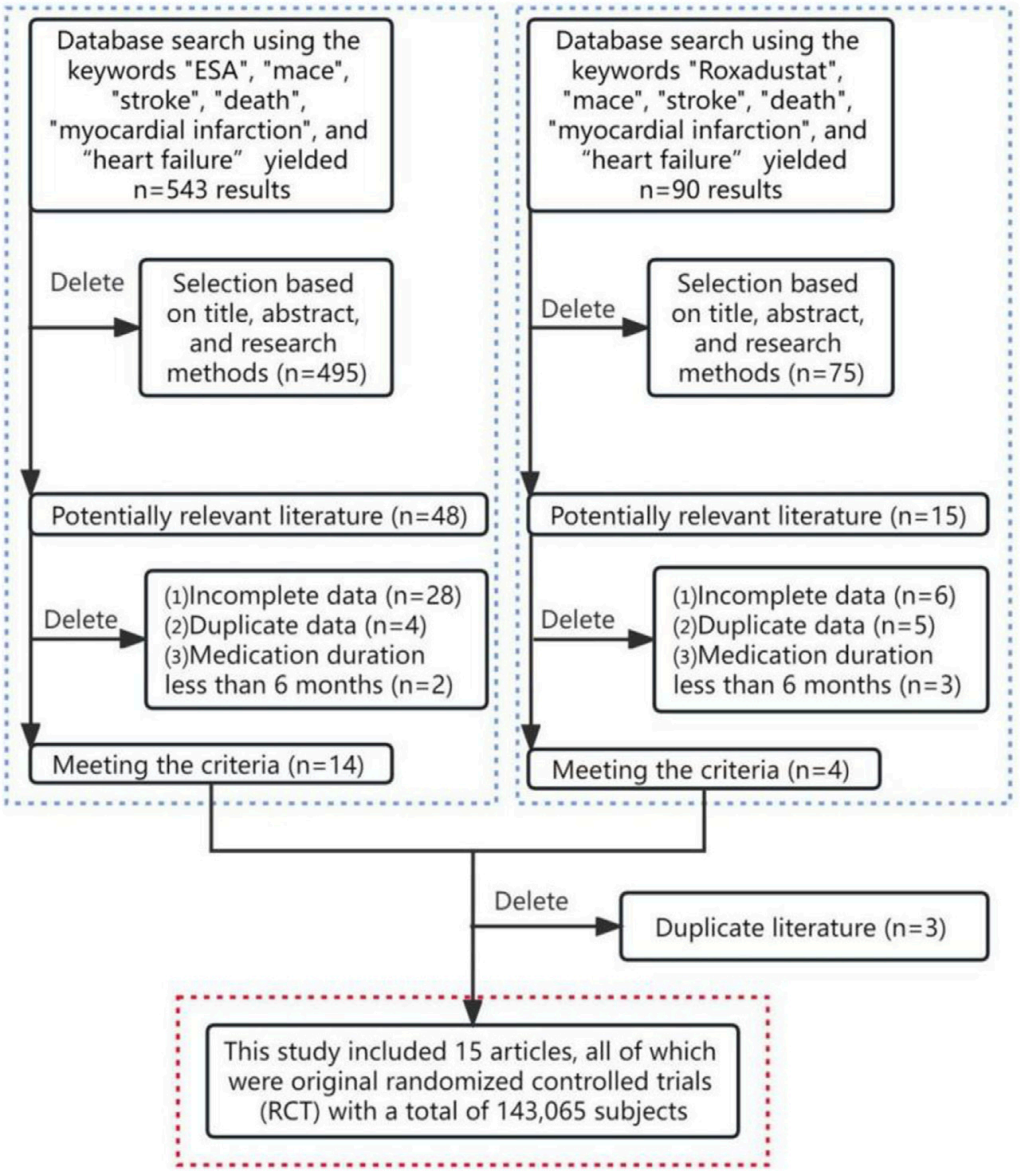
Flowchart of the literature screening process.

**FIGURE 2:**
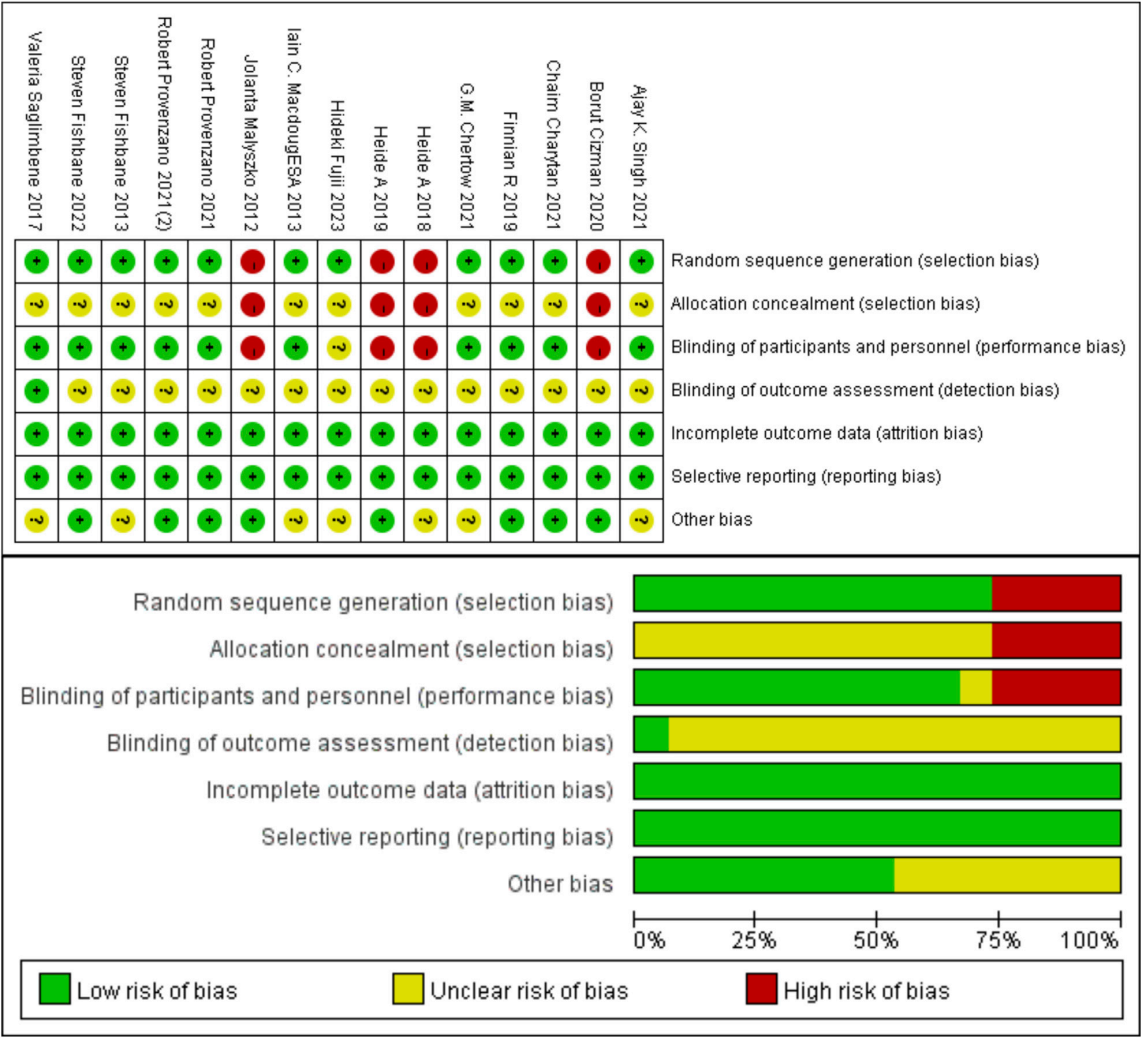
Risk-of-bias summary of included randomized trials using the Cochrane Risk-of-Bias Tool.

After removing duplicates, this study identified 633 potentially relevant articles (Figure 1). Exclusion of 567 articles based on title, abstract, and research methods left 66 articles for full-text evaluation. Of these, 48 were excluded for reasons including incomplete data, duplicate data, and medication duration not meeting the inclusion criteria. Additionally, three poster-format articles were excluded due to poor credibility and potential data duplication risks. Ultimately, 15 articles met the inclusion criteria (Fishbane et al., 2013; Macdougall et al., 2013; Malyszko et al., 2014; Saglimbene et al., 2017; Stirnadel-Farrant et al., 2018; Mc Causland et al., 2019; Stirnadel-Farrant et al., 2019; Cizman et al., 2020; Provenzano et al., 2021a; Provenzano et al., 2021b; Charytan et al., 2021; Chertow et al., 2021; Singh et al., 2021; Fishbane et al., 2022; Fujii et al., 2023). A keyword search for “Roxadustat” yielded three articles from phase 2 and phase 3 clinical trials in Chinese populations; however, three were removed for insufficient description of specific cardiovascular events (Chen et al., 2017; Chen et al., 2019a; Chen et al., 2019b). The analysis included 20 study cohorts with 1,43,065 patients, comprising 1,38,739 in the ESA treatment group and 4,326 in the Roxadustat group. All included articles were in English, and the quality assessment indicated no high-risk bias in the included literature (Figure 2).

#### 3.1.2 Studies’ features

The basic characteristics of the study cohorts are presented in Table 1. Of the 20 cohorts, 16 belonged to the ESA group—with 5 using Epoetin Alfa, 3 using Darbepoetin Alfa, 1 using CERA (Continuous Erythropoiesis Receptor Activator), 1 cohort using both Epoetin Alfa and Darbepoetin Alfa, and 6 unspecified. The Roxadustat group included four cohorts. Geographically, the ESA group had 2 cohorts from Asia, 5 from Europe, and 8 from the Americas, while all 4 Roxadustat cohorts were from the Americas. The studies reported consistent cardiovascular events in both the Roxadustat and ESA groups among the Chinese population with chronic kidney disease, though these were not analyzed in detail due to insufficient event descriptions (Chen et al., 2017; Chen et al., 2019a; Chen et al., 2019b). The cardiovascular event data for both treatment groups are shown in Table 2.

**TABLE 1:** Basic characteristics of included study cases.

Studies	Region	Sample size	Drug name	Follow-up time (months)	GFR (mL/min/1.73 m^2^)	Dialysis status	Male proportio*n* (%)	Average age (years)	History (%)		Hemoglobin level (g/dL)
									Cardiac disease	Cerebrovascular disease	
Chertow et al. (2021)	NA	1,732	Darbepoetin alfa	32	22.30 ± 12.00	0	57.30	65.70 ± 13.60	46.90	NA	9.78 ± 1.10
Mc Causland et al. (2019)	America	286	Darbepoetin alfa	6	15.00[11.00–21.00]	0	53.00	65.00 ± 10.50	35.60	19.10	11.30 ± 1.60
Fujii et al. (2023)	Asia	3,993	CERA	18	<15	1	63.70	65.50 ± 11.90	52.10	NA	10.70 ± 1.00
Cizman et al. (2020)	America	1,10,719	Epoetin Alfa	24	<15	1	47.40	63.09 ± 14.73	38.40	NA	10.60[9.50–11.70]
Fishbane et al. (2013)	Europe	542	Epoetin Alfa	36	<15	1	54.75	58.05 ± 13.70	35.25	NA	11.25 ± 0.55
Macdougall et al. (2013)	America	327	Darbepoetin alfa	36	<60	0	38.55	66.80 ± 14.64	38.20	NA	10.05 ± 0.65
Singh et al. (2021)	America	1,477	Epoetin Alfa or Darbepoetin alfa	30	<15	1	57.30	59.00(47.00–68.00)	45.00	7.40	10.39 ± 0.98
Malyszko et al. (2014)	Europe	398	ESA	12	<15	1	58.00	57.50 ± 14.70	NA	NA	11.04 ± 1.39
Stirnadel-Farrant et al. (2018)	Europe	109	ESA	24	<60	0	36.70	78.90 ± 11.50	50.30	25.40	10.40 ± 1.00
Saglimbene et al. (2017)	Europe	656	ESA	12	<15	1	61.60	61.46 ± 15.00	NA	4.40	11.00 ± 1.00
Stirnadel-Farrant et al. (2019)	America	3,893	ESA	72	<15	1	NA	NA	NA	NA	NA
Stirnadel-Farrant et al. (2019)	Europe	8,746	ESA	72	<15	1	NA	NA	NA	NA	NA
Stirnadel-Farrant et al. (2019)	Asia	3,921	ESA	72	<15	1	NA	NA	NA	NA	NA
Provenzano et al. (2021b), Provenzano et al. (2021a)	America	2,386	Roxadustat	12	<60	0	41.00	62.00 ± 14.00	NA	NA	9.10 ± 0.70
Provenzano et al. (2021b), Provenzano et al. (2021a)	America	522	Roxadustat	55	<15	1	59.20	53.80 ± 14.70	31.60	7.90	8.40 ± 1.00
Provenzano et al. (2021b), Provenzano et al. (2021a)	America	517	Epoetin Alfa	55	<15	1	58.90	54.30 ± 14.60	32.40	8.30	8.50 ± 1.00
Fishbane et al. (2022)	America	1,048	Roxadustat	42	<15	1	59.50	53.50 ± 15.30	20.90	8.50	10.20[9.30–10.90]
Fishbane et al. (2022)	America	1,053	Epoetin Alfa	42	<15	1	59.30	54.50 ± 15.00	21.90	7.60	10.30[9.20–11.00]
Charytan et al. (2021)	America	370	Roxadustat	12	<15	1	50.50	57.60 ± 13.60	35.90	11.10	10.30 ± 0.66
Charytan et al. (2021)	America	370	Epoetin Alfa	12	<15	1	58.00	58.40 ± 13.30	33.40	10.20	10.31 ± 0.66

Note: ⑴ Dialysis Status: 0 = Non-dialysis, 1 = Dialysis; ⑵ When only visual charts are available without numerical values, WebPlotDigitizer was used to extract the data; ⑶ For data conforming to a normal distribution, the mean ± standard deviation is presented, while for non-normally distributed data, the median [interquartile range] is presented; ⑷ CERA, continuous erythropoietin receptor activator; ⑸ NA, not available; ⑹ ESA, erythropoiesis-stimulating agent; ⑺ eGFR, estimated Glomerular Filtration Rate.

**TABLE 2:** Cardiovascular event situations in the ESA treatment group and roxadustat treatment group.

Studies	Region	Sample size	Drug name	Cardiovascular events				
				MACE				HF
				Death from any cause	MI	Stroke	Sum of MACE	
Chertow et al. (2021)	NA	1,732	Darbepoetin alfa	307	NA	NA	344	NA
Mc Causland et al. (2019)	America	286	Darbepoetin alfa	31	8	8	47	9
Fujii et al. (2023)	Asia	3,993	CERA	371	NA	NA	435	134
Cizman et al. (2020)	America	1,10,719	Epoetin Alfa	26,172	1,266	1,338	28,776	8,273
Fishbane et al. (2022)	Europe	542	Epoetin Alfa	64	29	20	113	49
Macdougall et al. (2013)	America	327	Darbepoetin alfa	22	11	3	36	28
Singh et al. (2021)	America	1,477	Epoetin Alfa or Darbepoetin alfa	233	126	35	394	73
Malyszko et al. (2014)	Europe	398	ESA	23	7	6	36	NA
Stirnadel-Farrant et al. (2018)	Europe	109	ESA	26	17	7	36	45
Saglimbene et al. (2017)	Europe	656	ESA	83	15	0	98	NA
Stirnadel-Farrant et al. (2019)	America	3,893	ESA	705	NA	NA	843	NA
Stirnadel-Farrant et al. (2019)	Europe	8,746	ESA	1,676	NA	NA	1,938	NA
Stirnadel-Farrant et al. (2019)	Asia	3,921	ESA	412	NA	NA	511	NA
Provenzano et al. (2021b), Provenzano et al. (2021a)	America	2,386	Roxadustat	400	86	56	480	175
Provenzano et al. (2021b), Provenzano et al. (2021a)	America	522	Roxadustat	2	6	8	16	5
Provenzano et al. (2021b), Provenzano et al. (2021a)	America	517	Epoetin Alfa	7	11	8	26	7
Fishbane et al. (2022)	America	1,048	Roxadustat	167	39	14	220	24
Fishbane et al. (2022)	America	1,053	Epoetin Alfa	187	41	12	240	29
Charytan et al. (2021)	America	370	Roxadustat	1	31	4	36	23
Charytan et al. (2021)	America	370	Epoetin Alfa	4	26	5	35	23

Note: ⑴ NA, not available; ⑵ MACE, major adverse cardiovascular events; ⑶ MI, myocardial infarction; ⑷ HF, heart failure.

#### 3.1.3 MACE event analysis in the ESA and Roxadustat groups

**FIGURE 3:**
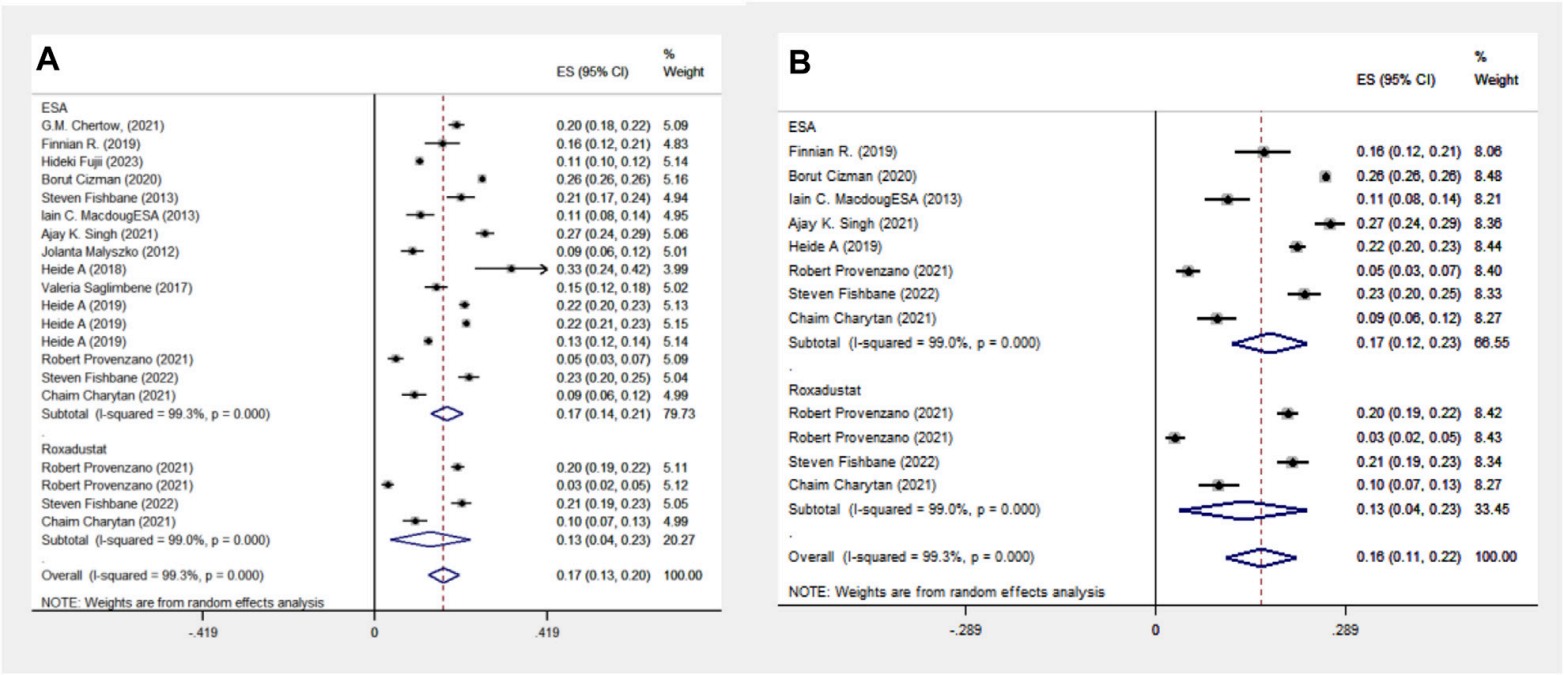
Overall group **(A)** and subgroup **(B)** forest plot of MACE events in the ESA and roxadustat groups.

The ESA group consists of studies employing Epoetin Alfa, Darbepoetin Alfa, CERA, or unspecified ESA formulations, including 16 cohort studies involving 1,38,739 patients. The Roxadustat group comprises four cohort studies with 4,326 patients. Meta-analysis shows no significant difference in MACE events between the groups, with a lower incidence in the roxadustat group (13% vs. 17%). Subgroup analysis for MACE events was limited to populations from the Americas due to the absence of cohort studies on Asian and European populations. The ESA group includes data from eight cohort studies and 118,642 patients, whereas the Roxadustat group consists of four cohort studies encompassing 4,326 patients. The meta-analysis reveals no statistically significant difference in MACE (Major Adverse Cardiac Events) events between the ESA and roxadustat groups. However, there is a lower occurrence of MACE events in the roxadustat group compared to the ESA group (13% vs. 17%), as illustrated in Figure 3.

#### 3.1.4 MI event analysis in the ESA and Roxadustat groups

**FIGURE 4:**
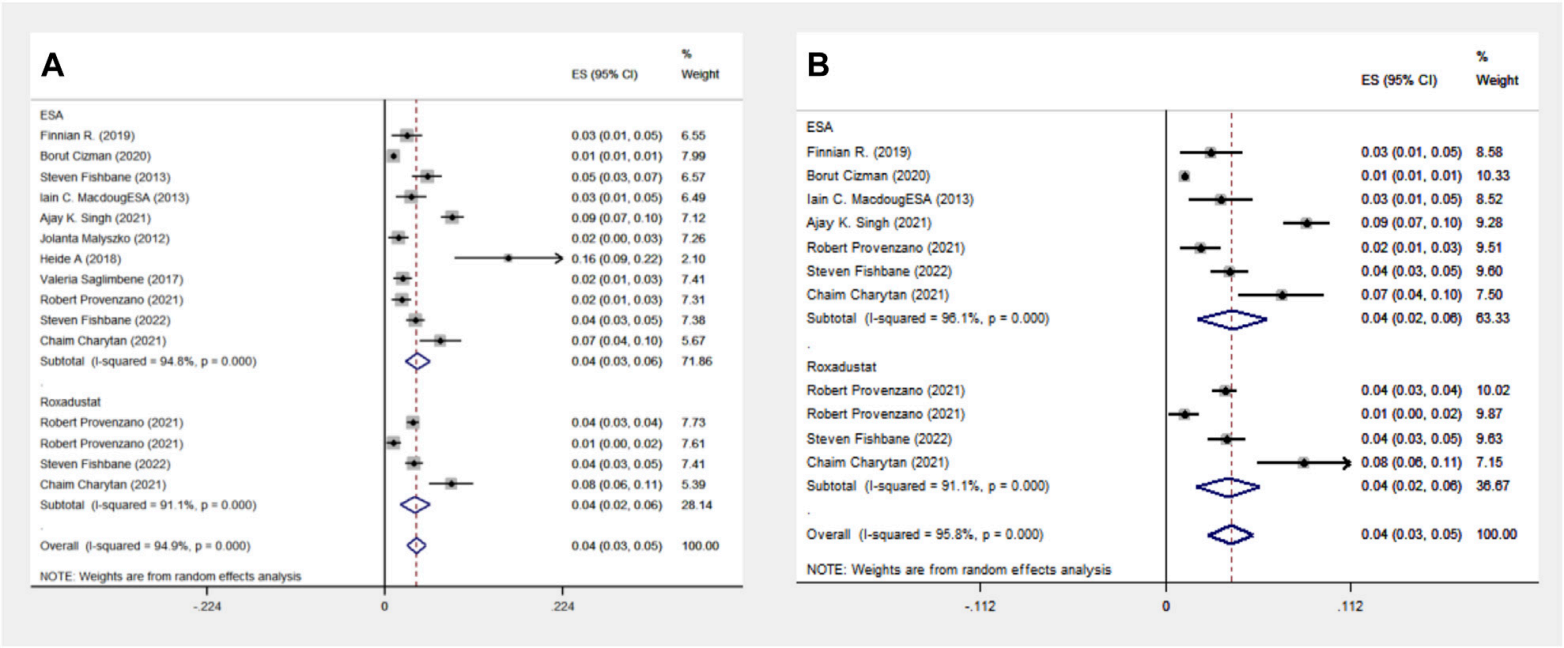
Overall group **(A)** and Subgroup **(B)** forest plot of MI events in the ESA and roxadustat groups. Abbreviations: MI, myocardial infarction.

The ESA group includes 11 cohort studies involving 1,16,454 patients, and the Roxadustat group includes four cohort studies with 4,326 patients. Meta-analysis reveals no significant difference in MI events between the two groups, each showing a 4% incidence rate. A subgroup analysis of the Americas confirmed this finding, with equal MI event probabilities in both groups, as indicated in Figure 4.

#### 3.1.5 Stroke event analysis in the ESA and Roxadustat groups

**FIGURE 5:**
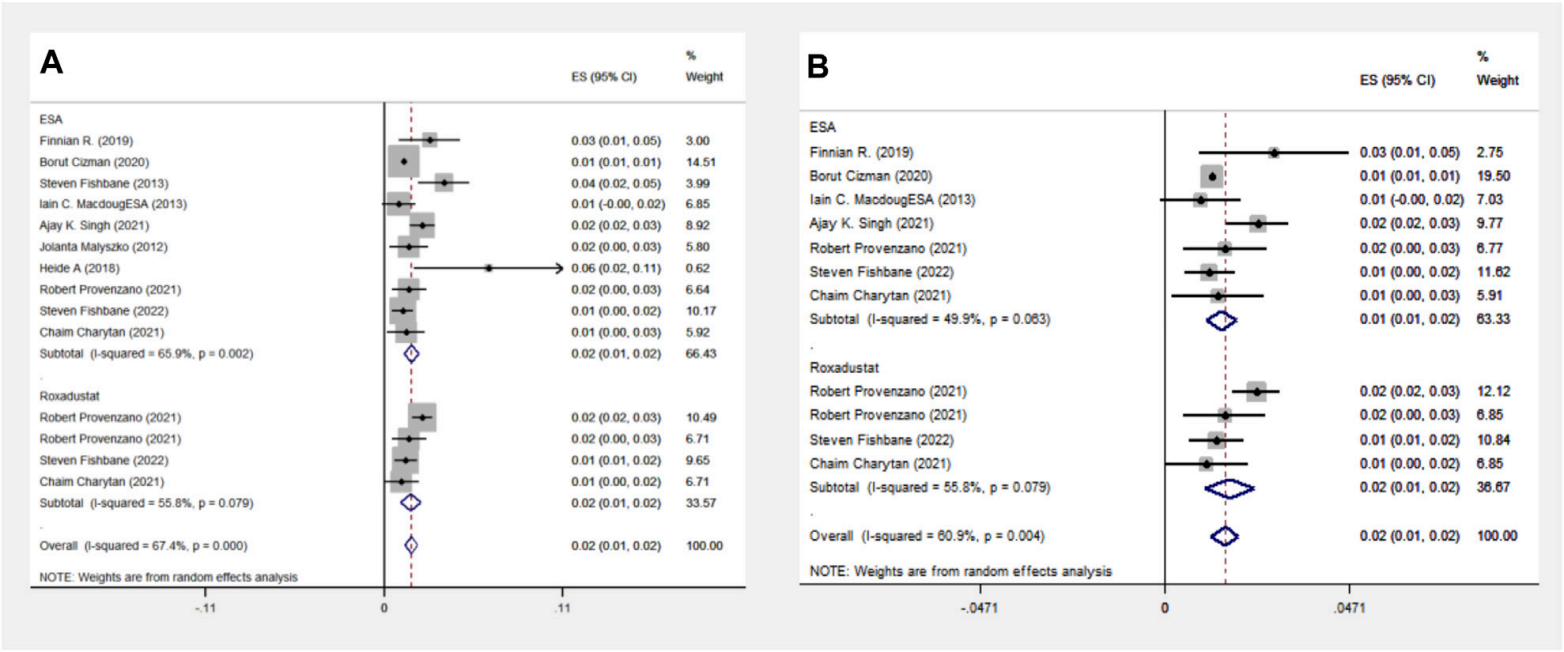
Overall group **(A)** and Subgroup **(B)** forest plot of stroke events in the ESA and roxadustat groups.

The ESA group comprises 10 cohort studies with data from 115,798 patients, whereas the Roxadustat group includes four cohort studies with 4,326 patients. Meta-analysis indicates no significant difference in stroke events between the groups, both reporting a 2% incidence rate. However, subgroup analysis in the Americas shows a slightly higher stroke event rate in the Roxadustat group compared to the ESA group (2% vs. 1%), as presented in Figure 5.

#### 3.1.6 Death event analysis in the ESA and Roxadustat groups

**FIGURE 6:**
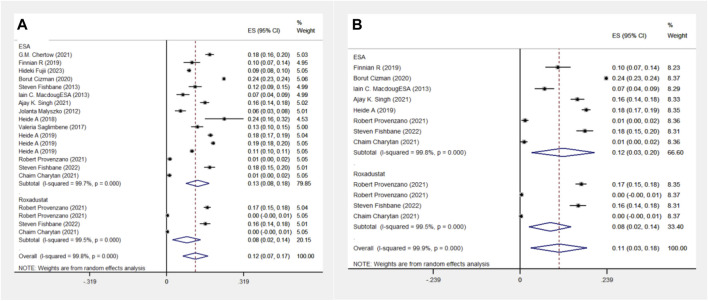
Overall group **(A)** and Subgroup **(B)** forest plot of death events in the ESA and roxadustat groups. Abbreviations: ESA, erythropoiesis-stimulating agent.

The ESA group consists of 16 cohort studies involving 1,38,739 patients, while the Roxadustat group comprises 4 cohort studies with 4,326 patients. The meta-analysis revealed no significant differences in death events between the two groups. However, the Roxadustat group showed a lower probability of death events compared to the ESA group (8% vs. 13%). A similar trend was observed in subgroup analysis for populations from the Americas, with the Roxadustat group also showing a lower incidence of death events than the ESA group (8% vs. 12%), as illustrated in Figure 6.

#### 3.1.7 Heart failure events analysis in the ESA and Roxadustat groups

**FIGURE 7:**
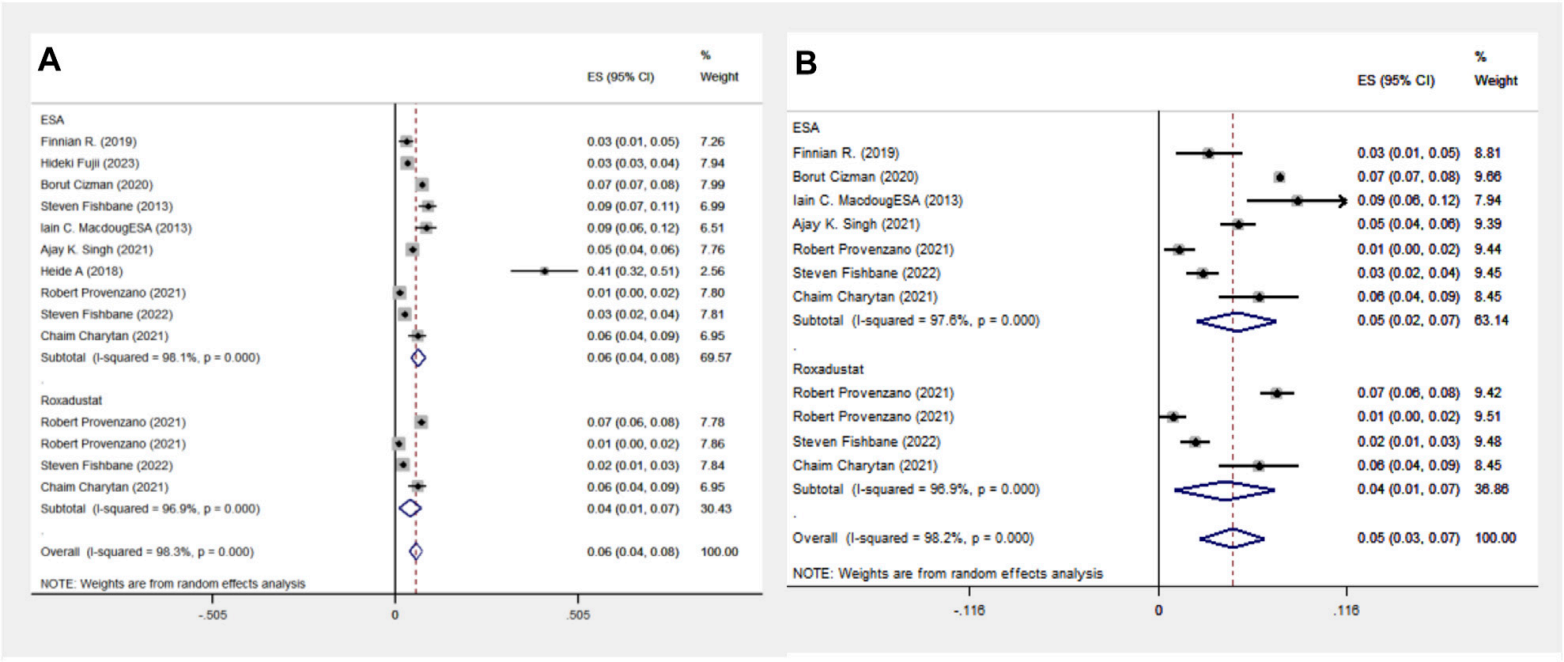
Overall group **(A)** and Subgroup **(B)** forest plots of HF events in the ESA and roxadustat groups.

In another comparison, the ESA group encompassed 10 cohort studies with data from 119,393 patients, and the Roxadustat group included 4 cohort studies with 4,326 patients. The meta-analysis showed no significant differences in heart failure (HF) events between the groups. The Roxadustat group had a lower probability of HF events compared to the ESA group (4% vs. 6%). Subgroup analysis for populations from the Americas mirrored these findings, showing a reduced incidence of HF events in the Roxadustat group compared to the ESA group (4% vs. 5%), as depicted in Figure 7.

#### 3.1.8 Bias testing in the study

**FIGURE 8:**
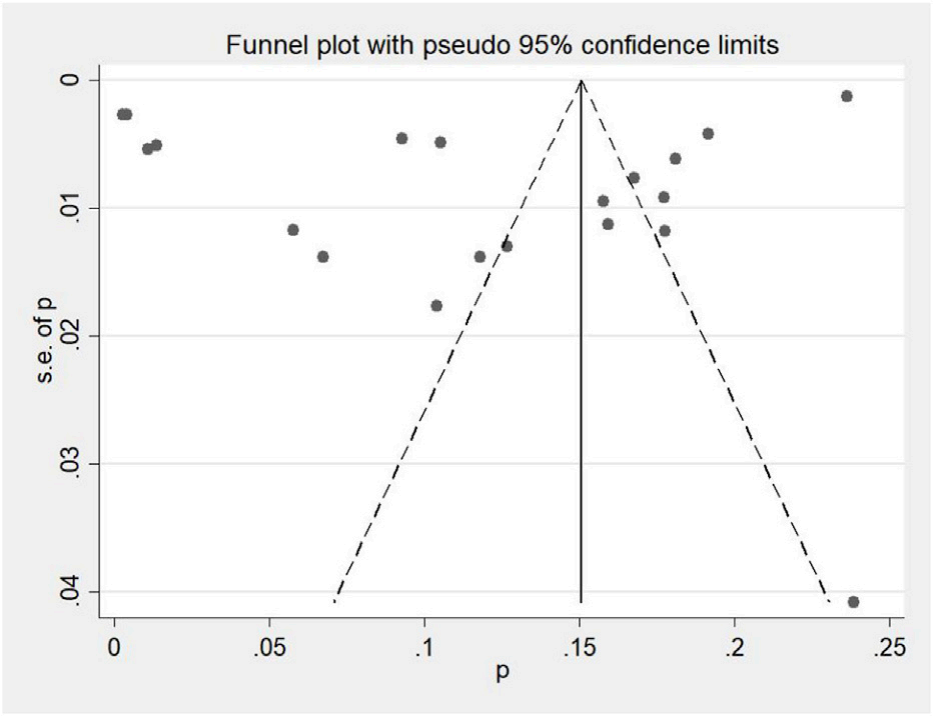
Funnel plot of the included study cohorts in the ESA group and the roxadustat group.

Overall, 15 articles met the inclusion criteria, covering 20 study cohorts and 1,43,065 patients. Figure 8 displays the effect points of each study, indicating minimal publication bias among the cohorts.

#### 3.1.9 Sensitivity analysis of study results

**FIGURE 9:**
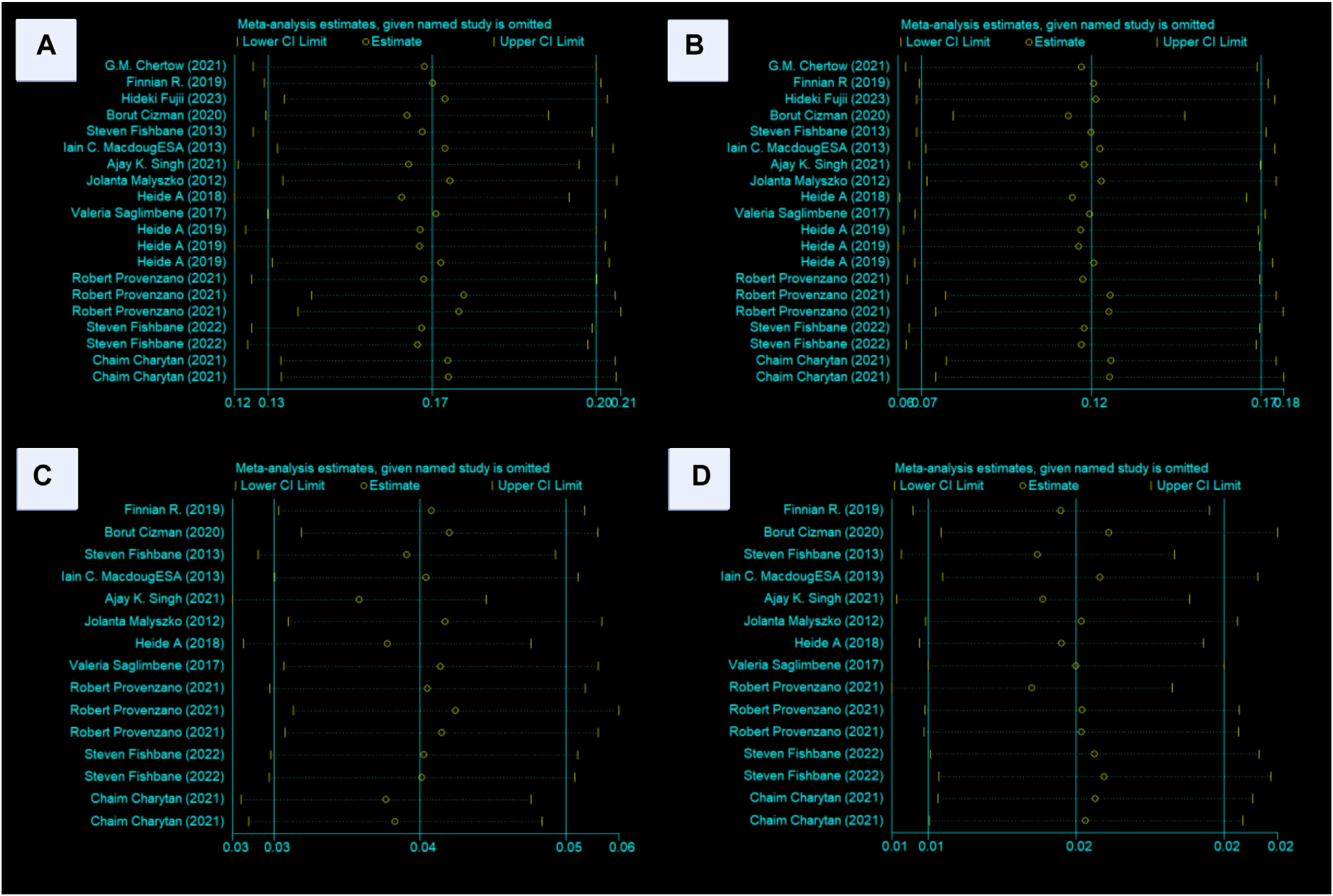
**(A**–**D)** Sensitivity analyses of the MACE, death, MI, and stroke conclusions. Abbreviations: MACE, major adverse cardiovascular events, MI, myocardial infarction, ESA, erythropoiesis-stimulating agent.

Sensitivity analysis using the one-by-one exclusion method revealed no significant heterogeneity in the investigated indicators. This suggests the stability and reliability of the study conclusions, as illustrated in Figure 9.

### 3.2 Bioinformatics analysis of the effects of Roxadustat on human cells

#### 3.2.1 Data source

The dataset GSE189905 was retrieved from the NCBI database using “Roxadustat” as the search term and “human (*H. sapiens*)” as the sample source. The control group included samples GSM5709349, GSM5709350, and GSM5709351, while samples GSM5709355, GSM5709356, and GSM5709357 comprised the experimental group.

#### 3.2.2 Data processing

The dataset GSE189905 contains RNAseq data on the effects of Roxadustat in human neuroblastoma cell lines. Differentially expressed genes were identified using the criteria of “*p* < 0.01 and a 2-fold logFC,” resulting in a total of 59 differentially expressed genes.

#### 3.2.3 Results of GO enrichment analysis, KEGG pathway analysis

**FIGURE 10:**
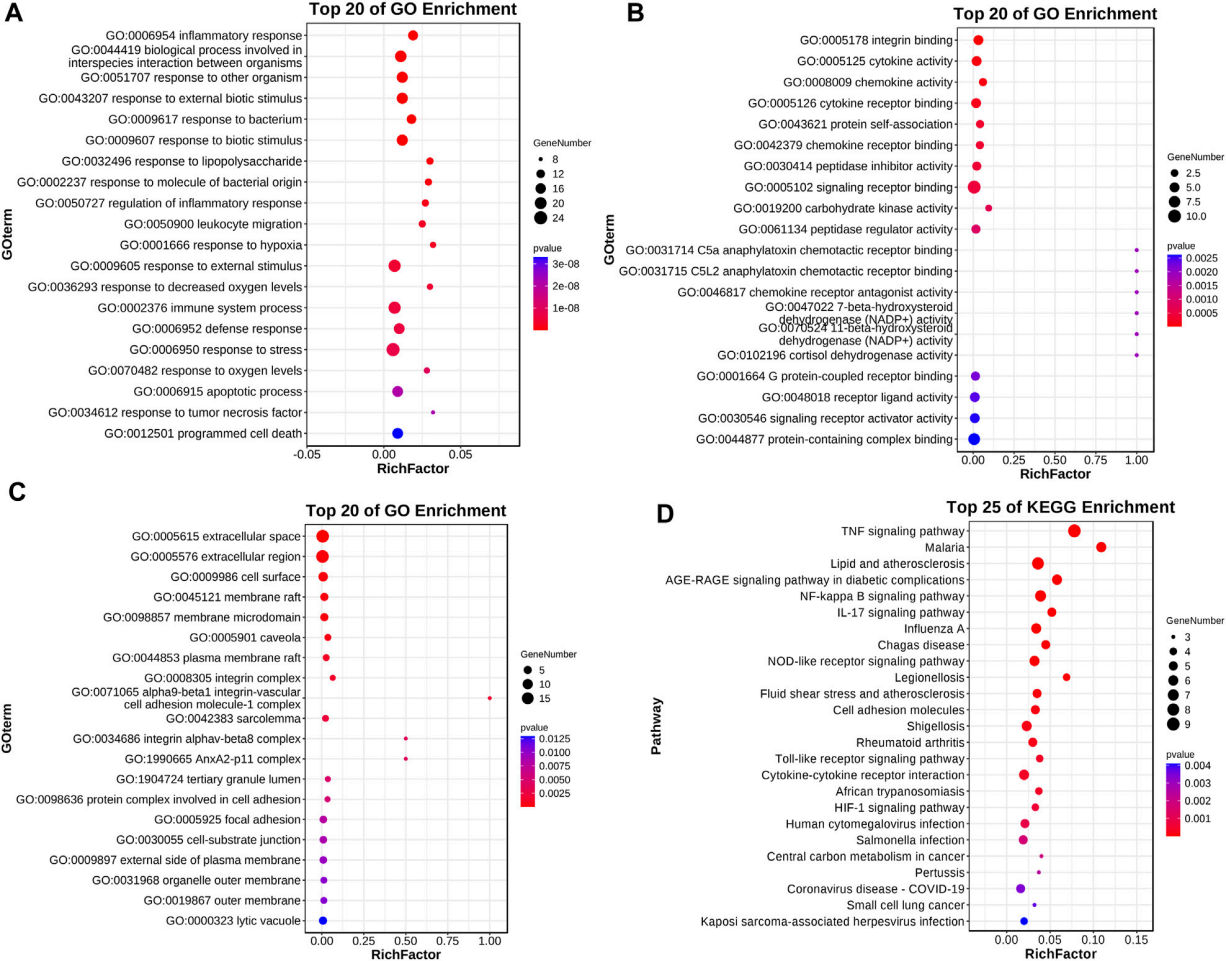
Roxadustat-related differential genes GO enrichment analysis and KEGG pathway analysis. Panel **(A)** displays the biological process enrichment, panel **(B)** shows the molecular function enrichment, panel **(C)** presents the cell component enrichment, and panel **(D)** illustrates the KEGG pathway analysis.

GO analysis revealed the impact of Roxadustat on biological processes, molecular functions, and cellular components of the neuroblastoma cell line. Notably, Roxadustat influences inflammatory responses (GO:0006954, *p*-value 4.8310-12, FDR 2.8810-9), integrin binding (GO:0005178, *p*-value 1.8510-4, FDR 2.3010-2), and primarily affects the extracellular space (GO:0005615, *p*-Value 7.8210-6, FDR 7.0410-4). KEGGs pathway analysis identified significant pathways such as the TNF signaling pathway (hsa04668, *p*-Value 1.3610-9, FDR 1.5610-7), NF-κB signaling pathway (hsa04064, *p*-Value 6.5810-7, FDR 2.5210-5), and pathways related to lipid metabolism and atherosclerosis (hsa05417, *p*-Value 3.2510-6, FDR 9.3310-5) that are closely associated with inflammation [38][39][40]. Results were visualized using the “ggplot” package in R, as shown in Figure 10.

#### 3.2.4 Results of PPI network

**FIGURE 11:**
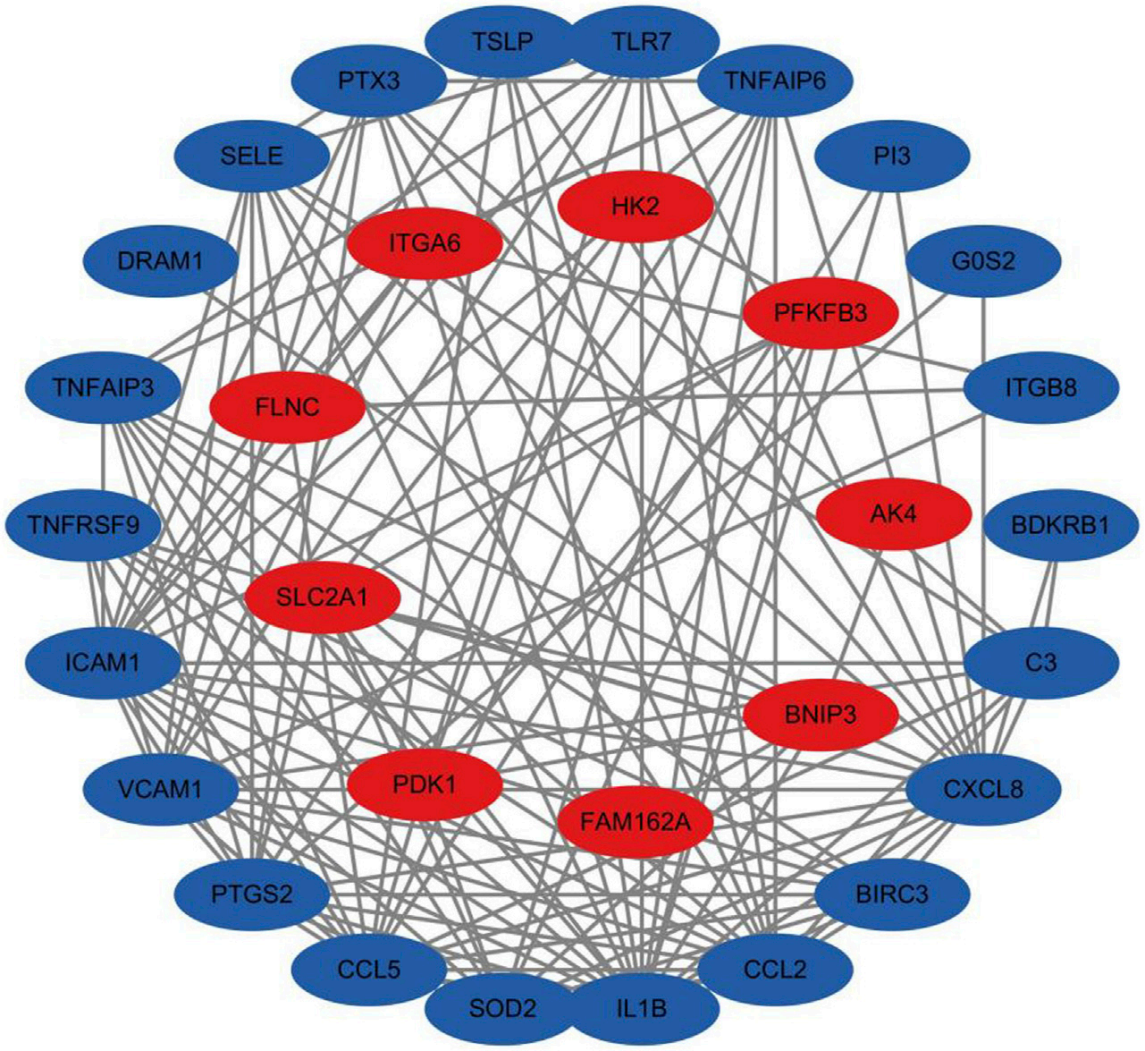
Protein interaction network analysis results, with red indicating genes of upregulated proteins and blue indicating genes of downregulated proteins.

The network comprises 36 nodes and 130 edges, with an average node degree of 7.22. This complexity suggests a dense protein-protein interaction network with extensive interactions among the proteins. The observed number of edges significantly exceeds the expected number of 27, illustrating that the protein interactions surpass random connectivity. The PPI enrichment *p*-value is below 1.0E-16, emphasizing the significance and abundance of these interactions, which supports the reliability of the findings. Notably, proteins such as IL1B and CXCL8 have a degree of 20, indicating their potential as key nodes in the network. Similarly, ICAM1 has a degree of 18, and both CCL2 and CCL5 have degrees of 16, highlighting their important roles as depicted in Figure 11. These proteins, known for their role in inflammatory responses, are downregulated, suggesting that Roxadustat may help mitigate inflammation.

## 4 Discussion

Anemia is a frequent issue in chronic kidney disease, and the traditional treatment, erythropoiesis-stimulating agents (ESAs), can enhance life quality and reduce blood transfusion needs. However, high doses of ESAs may heighten the risk of cardiovascular problems and hasten kidney failure (Koulouridis et al., 2013). When ESAs show low efficacy, increasing the dose to meet the desired hemoglobin levels could also amplify these risks (Solomon et al., 2010). Under normal oxygen conditions, hypoxia-inducible factor prolyl hydroxylase inhibitors (HIF-PHIs) mimic low-oxygen effects by inhibiting prolyl hydroxylase domain (PHD) activity. This triggers a cascade of gene expressions related to hypoxia adaptation, boosts endogenous erythropoietin (EPO) production, elevates hemoglobin levels, and ameliorates anemia. Furthermore, HIF-PHIs can enhance iron metabolism by lowering hepcidin levels, thereby increasing iron absorption and mobilizing stored iron (Schwartz et al., 2019). Roxadustat, the first HIF-PHI introduced globally, enhances EPO production within normal ranges, improves iron metabolism, and effectively treats anemia in chronic kidney disease (Haase, 2017). Nonetheless, as HIF upregulation affects various metabolic pathways—including those involved in angiogenesis, lipid and glucose metabolism, glycolysis, mitochondrial function, cell growth, survival, vasodilation, and cell migration—the cardiovascular safety of Roxadustat is under scrutiny. Studies have shown that Roxadustat has a lower incidence of cardiovascular composite events compared to traditional ESA treatments (12% vs. 17%) (Provenzano et al., 2016). A meta-analysis focused on cardiovascular events in the treatment of anemia with ESA and Roxadustat revealed that Roxadustat appears safer concerning major adverse cardiac events (MACE), death, and heart failure. This safety profile may be linked to how Roxadustat maintains EPO production within a physiological range, unlike ESAs, which are administered in intermittent high doses, leading to peak EPO levels and a higher risk of cardiovascular events (Szczech et al., 2010). Additionally, the high doses of ESA needed to reach desired hemoglobin levels are associated with increased mortality rates (Koulouridis et al., 2013). Roxadustat, by producing endogenous EPO levels close to the physiological norm, minimizes the risk of adverse events (Gupta and Wish, 2017). Compared to ESAs, Roxadustat has less impact on blood pressure, can ameliorate cholesterol issues, lower blood glucose, enhance insulin sensitivity, and mitigate cardiovascular risk factors (Flamme et al., 2014; Provenzano et al., 2016; Riopel et al., 2020). Additionally, Roxadustat may improve myocardial ischemia and reduce the area of myocardial infarction (Deguchi et al., 2020). Animal studies indicate that HIF is crucial in myocardial ischemic preconditioning, enhancing heart muscle adaptability (Bishop and Ratcliffe, 2015).

In the absence of a database for Roxadustat in cardiovascular disease-related models, samples for this study were obtained from *H. sapiens* neuroblastoma cell lines, both with and without Roxadustat treatment. GO enrichment analysis revealed that Roxadustat primarily impacts the molecular function associated with integrin binding (GO:0005178), affecting genes such as ICAM1, VCAM1, IL1B, ITGB8, and ITGA6. These genes, which are linked to endothelial cells and inflammation, were identified using sources like The Human Gene Database (https://www.genecards.org/) and NCBI gene summaries. Roxadustat’s biological processes predominantly relate to the inflammatory response (GO:0006954). Integrins are crucial in endothelial and antigen-presenting cells within immune inflammation contexts (Takada et al., 2007), which are vital components of the vascular barrier and relate to cardiovascular events. Consequently, it is hypothesized that Roxadustat may influence endothelial cells and inflammatory responses, potentially impacting cardiovascular events, though further validation is needed. KEGG pathway analysis highlighted three significant pathways: the TNF signaling pathway, NF-κB signaling pathway, and the lipid and atherosclerosis pathway—all of which are closely associated with inflammation. The TNF signaling pathway is essential for regulating the inflammatory response (Yu et al., 2020). External stimuli cause cells to produce TNF-α, which activates several signaling pathways, including NF-κB, MAPK, and PI3K, leading to a cascade of biological effects. TNF-α specifically activates the NF-κB pathway, which promotes inflammatory factor expression in the nucleus (Hoesel and Schmid, 2013). Moreover, there is a strong link between lipid metabolism, atherosclerosis (Warnatsch et al., 2015; Libby, 2021), and inflammation. Protein interaction network analysis suggests that Roxadustat may help alleviate inflammation.

Hypoxia signaling is linked to calcification. Research using rodent models both *in vivo* and *in vitro* demonstrates that the hypoxia pathway may enhance vascular calcification (Mokas et al., 2016). HIF-1, a heterodimeric transcription factor, consists of an unstable α subunit (HIF-1α) and a stable β subunit (HIF-1β). In CKD mouse models, Roxadustat and Daprodustat have been shown to accelerate phosphate-induced calcification through activation of the HIF-1 pathway. However, extensive clinical studies are necessary to determine if these drugs heighten the risk of vascular calcification in CKD patients (Mokas et al., 2016; Tóth et al., 2022). Observational studies indicate that serum HIF-1α levels can predict vascular calcification in type 2 diabetes patients (Li et al., 2014). The 2020 guidelines from the Asia Pacific Society of Nephrology suggest that HIF-PHIs are linked with thrombosis and vascular calcification. Patients with a history of these conditions should exercise caution when using these drugs, and further studies are required to clarify the association between HIF-PHIs and these adverse outcomes (Yap et al., 2021).

Our study meticulously examined its limitations. The cohorts included, whether treated with ESA or Roxadustat, had varied drug dosages and study designs, potentially affecting the results due to inconsistency. The age, gender distribution, and comorbidities also varied across the cohorts, possibly influencing the outcomes due to heterogeneity. Moreover, no database exists for Roxadustat concerning cardiovascular disease in cell lines or animal models. We utilized biological samples from human neuroblastoma cell lines related to Roxadustat, which might have impacted data quality. Future *in vivo* or *in vitro* studies are encouraged to confirm the reliability of our findings. Despite these limitations, our sensitivity analysis did not reveal significant heterogeneity, and efforts were made to identify databases aligned with our research objectives. Overall, our study provides a deeper investigation into the cardiovascular safety of Roxadustat, aiming to aid in the development of improved clinical treatment strategies.

## 5 Conclusion

A meta-analysis of cohorts in the general and American populations showed no significant differences in cardiovascular event rates between patients treated with Roxadustat and ESA for anemia associated with chronic kidney disease. However, the Roxadustat group appears to exhibit a safer profile concerning major adverse cardiovascular events (MACE), death, and heart failure. Bioinformatics analysis indicates that Roxadustat may influence integrin adhesion and affect the TNF and NF-κB signaling pathways, along with lipid and atherosclerosis pathways, potentially reducing inflammation.

## Data Availability

The original contributions presented in the study are included in the article/Supplementary material, further inquiries can be directed to the corresponding author.
